# Non-Familial Cherubism with Bilateral Maxilla and Mandible Involvement – Clinicoradiographic Findings

**DOI:** 10.7759/cureus.709

**Published:** 2016-07-25

**Authors:** Ibrahim K Ali, Freny R Karjodkar, Kaustubh Sansare, Prashant Salve, Amaresh C Dora

**Affiliations:** 1 Oral Medicine and Radiology, Nair Hospital Dental College

**Keywords:** cherubism, bilateral swelling, radiolucency

## Abstract

Cherubism is a self-limiting non-neoplastic autosomal dominant fibro-osseous syndrome of the jaws. It is occasionally manifested before the age of two years. It occurs in children and more often in boys. It is characterized by notable clinical bilateral swelling of the cheeks due to a bony enlargement of the jaws that impart a characteristic ‘cherubic’ look. Regression occurs in the course of puberty leaving a few facial deformities and malocclusion. Cherubism might occur in solitary cases or in several members of the family, often in many generations. The reported case is an example of solitary sporadic occurrence within a family, which is a rarely documented condition in the literature.

## Introduction

Cherubism is a rare, inherited disease of children which is characterized by bilateral painless mandibular and often maxillary swelling that results in a fullness of cheeks, firm protuberant intraoral alveolar mass, and displaced or missing teeth. Cherubism is inherited with a penetrance of almost 100% in males and nearly 50-75% in females [1]. Cherubism was first described by William Jones in 1933 as a familial multilocular cystic disease of the jaws accompanied by swelling of the cheeks [2]. It is a rare, benign, genetically inherited osteoclastic fibro-osseous lesion. Maxillary involvement might result in slight upward turning of the eyes revealing some abnormal amount of sclera. Expansion of the maxilla can cause retraction of the lower eyelid, stretching of the skin, and an inverted v-shaped palatal arch. Cervical lymphadenopathy is usually present in early stages due to reactive hyperplasia and fibrosis though may subside by puberty [1]. Cherubism has been categorized depending on the severity of condition according to the Seward and Hankey system [3]. In the majority of reported cases, the lesions are restricted to the mandible, with the condyles being spared. In the case reported here of a 15-year-old female, both mandible and maxillae with the coronoid process were involved, and no significant family history was found.

## Case presentation

**Figure 1:**
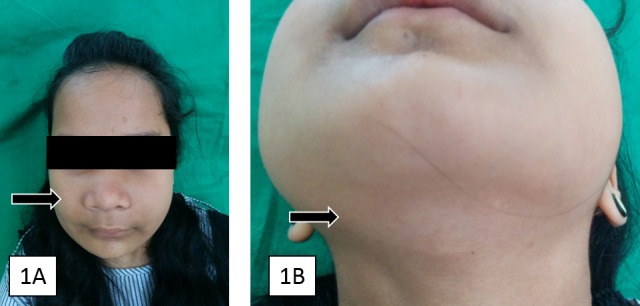
Frontal View (A) Bilateral swelling of the cheek with characteristic cherubic look due to the enlargement of maxillary and mandibular ridges,  (B)  Enlarged right submandibular lymph node.

A 15-year-old female patient reported to the Oral Medicine unit with complaint of bilateral swelling of the face for the past three years. The swelling started initially as smaller in size at nine years of age, which gradually increased in size to the present size. The patient agreed to participate and was explained the nature and objectives of this study, and informed consent was formally obtained. No reference to the patient's identity was made at any stage during data analysis or in the report. Family history did not reveal any previous records regarding cherubism in the other members of the family. On extra-oral examination, facial asymmetry was identified (Figure 1A). Diffuse, bilateral swelling was noticed over the face measuring about 4 cm × 4 cm in dimensions. Skin over the swelling was normal, freely movable, and intact. The right submandibular lymph node was enlarged, palpable, and non-tender (Figure 1B).

**Figure 2:**
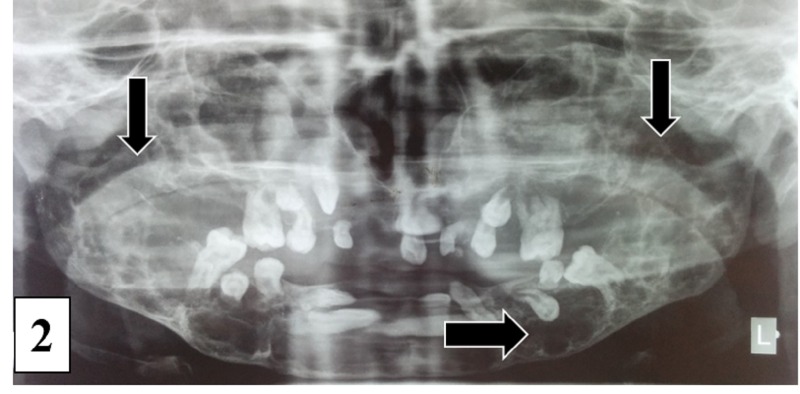
Panoramic Radiograph Multiple radiolucencies involving the maxilla and mandible bilaterally, unerupted and displaced permanent teeth along with involvement of bilateral coronoid process.

The teeth present were displaced from their normal position, with multiple missing teeth in mandible and maxilla. Expansion of the buccal and lingual cortical plate was noticed, and the overlying mucosa was normal. Laboratory investigations showed an elevated serum alkaline phosphatase value of 188 IU/L (normal, 0 to 150 IU). Serum calcium and phosphorus levels and other investigations were within normal limits. Panoramic radiograph revealed multiple radiolucencies involving the bilateral maxilla and mandible along with the coronoid process and unerupted and displaced permanent teeth (Figure 2).

**Figure 3:**
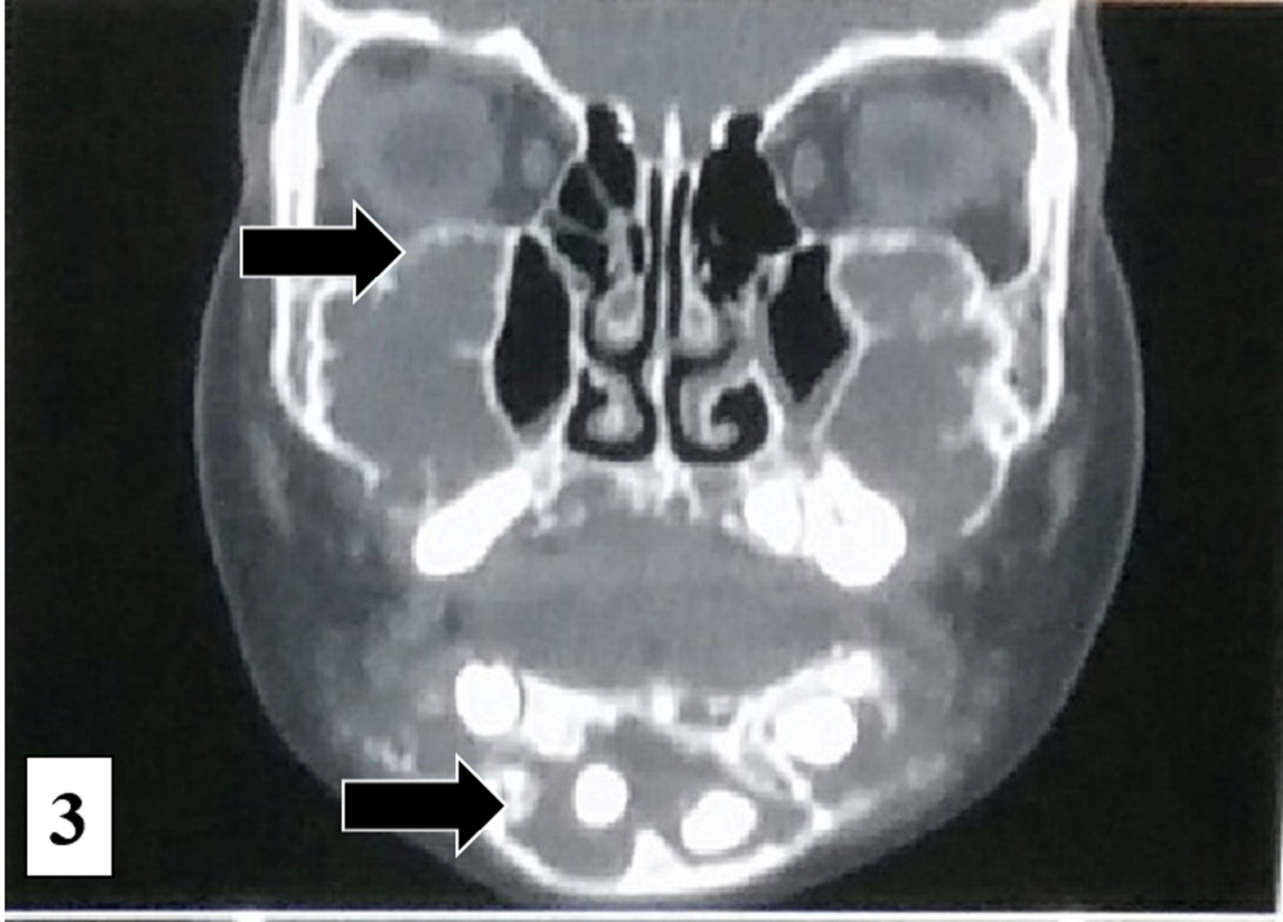
Computed Tomography in the Coronal Section A large, extensive, lytic lesion with soft-tissue density involving the entire mandible, maxilla, and lifting of the orbital floor.

Computed tomography showed a well-defined multilocular radiolucent lesion resulting in the thinning and expansion of the buccal and lingual cortical plates, involving the entire mandible, maxilla, and lifting of the floor of the orbit (Figure 3).

**Figure 4:**
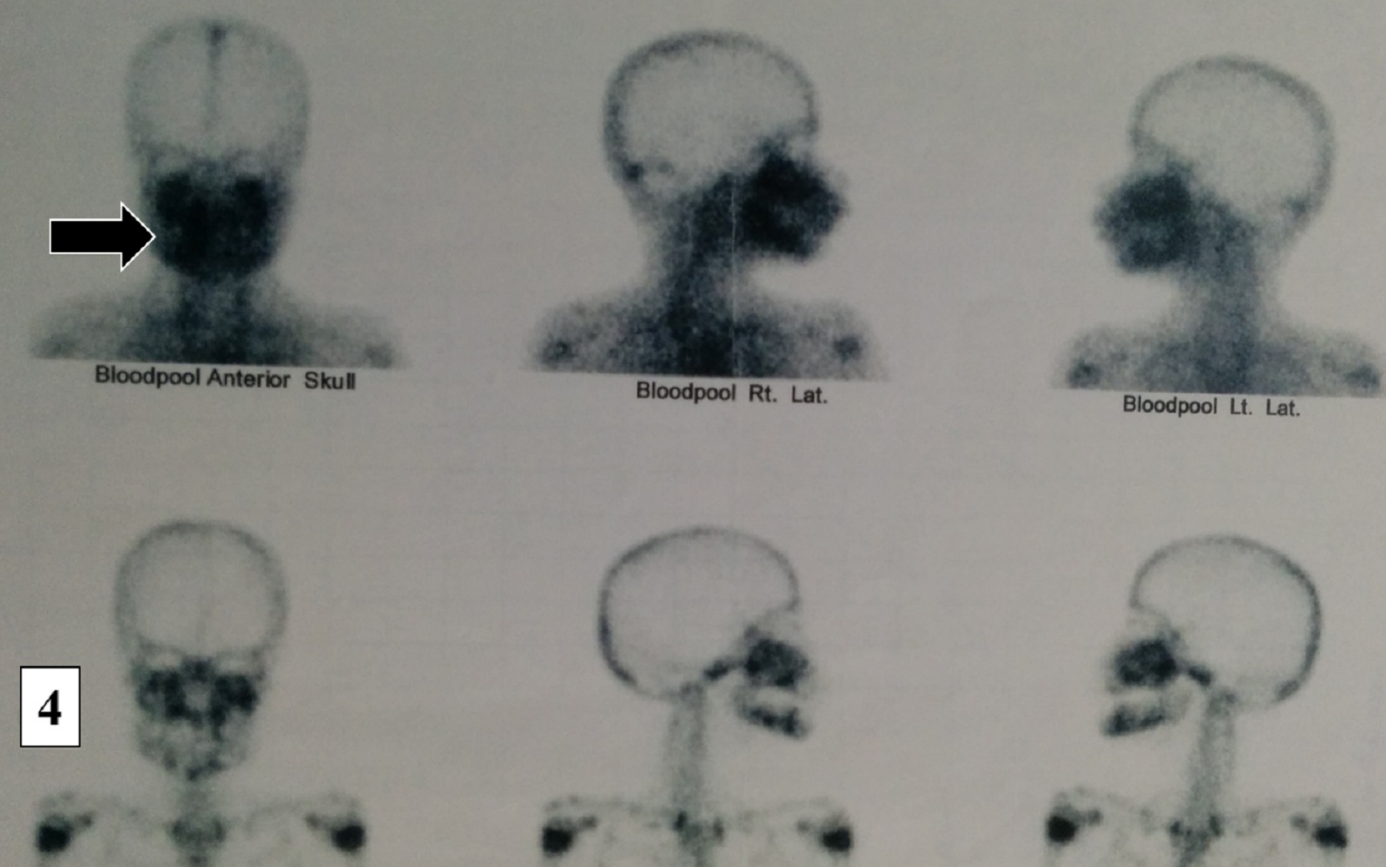
Three-Phase Bone Scan and Blood-Pool Images Increased vascularity in the region of the mandible and maxilla suggestive of infective or inflammatory pathology.

A three-phase bone scan was done with 10 mCi of 99MTc-MDP injected IV and blood-pool images were taken immediately. Static images were taken three hours later. Blood-pool images revealed increased vascularity in the region of the mandible and maxilla, suggestive of an infective or inflammatory pathology. A static whole body bone scan showed increased radiotracer uptake in the maxilla and mandible (Figure 4). Physiological tracer uptake was seen in the upper end of the right and left humerus.

## Discussion

Clinical or radiographic features of cherubism are not noticeable prior to fourteen months to three years of age. Characteristically, the earlier the lesion appears, the more abruptly it progresses. This patient first reported clinical swelling at the age of nine years. The progressive swelling of the face with marked increase in fullness of the cheeks is due to expansion of underlying bony structures. The bilateral enlargement of the maxilla contributes to cherubic analogy by exposing sclera causing ‘eyes raised to heaven’ look. In the present case, sclera was not prominently exposed, and hence, the ‘eyes raised to heaven’ feature was not noticeable. Radiologically it shows peculiar bilateral multilocular cystic expansion of the jaws. The presence of unerupted teeth and the destruction of the alveolar bone may cause displacement of teeth, resulting in a ‘floating tooth syndrome’ appearance [4]. In our patient, panoramic radiograph showed multilocular osteolytic lesion involving the entire body and ramus of the mandible and maxilla, sparing the condyle with floating tooth appearance. The radiographic findings of cherubism on CT are expansive bone cortex thinning, remodeling, multilocular radiolucency with a coarse trabecular pattern, dental anomalies, and absence of periosteal reaction. Sparing of the condyles has been described as a characteristic feature of cherubism although a few cases of patients with condylar involvement have been reported in the literature. Hence, a finding of condylar involvement should not exclude the diagnosis of cherubism if other characteristic features are present within the jaw. Based on radiographic features, a provisional diagnosis of cherubism and differential diagnosis of fibrous dysplasia, brown tumor in hyperparathyroidism, familial gigantiform cementoma, Jaffe-Campanacci syndrome, and odontogenic cyst were established. Precise features for the diagnosis of cherubism include bilateral mandibular involvement, limitation to the maxilla and mandible, and regression at the time of puberty. Brown tumor and Jaffe-Campanacci syndrome are distinguished from cherubism on clinical findings. Familial gigantiform cementoma is an uncommon, osseous disorder characterized by the production of cementum within the lesion. Bilateral odontogenic cysts are rare during the first five years of life [5]. Seward and Hankey (1957) suggested a grading system for cherubism: Grade I: involvement of bilateral mandibular molar regions and ascending rami, mandible body, or mentis; Grade II: involvement of bilateral maxillary tuberosities (in addition to grade 1 lesions) and diffuse mandibular involvement; Grade III: massive involvement of the entire maxilla and mandible, except the condyles; and Grade IV: involvement of both jaws, including the condyles [3].

This patient was classified as Grade III subtype of cherubism with involvement of the coronoid process. Multiplane and three-dimensional CT imaging elucidate the accurate extent of the disease. Magnetic resonance imaging (MRI) features of cherubism described in the literature are nonspecific, but it is useful in clarifying the extent, particularly proximity of the cherubic lesions to the orbits and the optic nerves [5-6]. In our case, no sign or symptoms were present to indicate an MRI investigation. Cherubism is similar to fibrous dysplasia in terms of radiographic features and has been formerly described as a familial fibrous dysplasia. Nevertheless, genetic analysis has shown that the cherubism is caused by different mutations than subtypes of fibrous dysplasia (monostotic, polyostotic, craniofacial and McCune-Albright syndrome) and is nowadays considered as a distinct disease at the molecular level [5, 7]. Cherubism may be present in alliance with other genetic diseases such as Ramon’s syndrome or Noonan’s syndrome (pseudo-Turner syndrome) [8]. Radiological findings are diagnostic, and diagnosis can rely on distinctive radiological features alone. Meanwhile, we could not implement a genetic analysis in our patient; we are not able to discuss the existence of a potential specific mutation. Cherubism is associated with an unusual pattern of teeth eruption, teeth agenesis, premature loss of deciduous teeth, enamel hypoplasia, and the presence of ectopic teeth. Cervical lymph nodes are routinely involved during active phase of the disease and are non-tender on palpation, mobile, and discrete [4, 9]. In this patient, cervical lymph nodes were involved, enlarged, and non-tender on palpation. No such similar findings were reported in our patient. Progression of orbital involvement rarely continues beyond puberty after stabilization or regression of the lesion. Surgical intervention in the active phase of disease is usually not recommended since it is a self-limiting condition and has a tendency to regress after puberty. Radiation therapy is contraindicated in cherubism due to the risk of post-radiation sarcoma and growth disturbances [10]. Calcitonin acts as an anti-resorptive agent known to cause regression by inhibition of bone resorption by osteoclast [8]. This case has not shown any significant progression since initial clinical examination. The patient is recalled every month and examined for any substantial facial expansion. In the present case, due to the self-limiting nature and active phase of the disease, there is no need to perform any surgery unless a complication develops.

## Conclusions

Cherubism is usually a self-limiting condition and regresses with age. Treatment depends on the clinical course of the disease and is suggested only in the cases of esthetic or functional needs. Most investigators preferably rely on waiting until the end of puberty before planning for any surgical intervention. Surgery is indicated only in aggressive cases with functional impairments like speech, chewing or swallowing, ocular disturbances, or with the presence of significant facial deformities that may affect the patient’s psychological state. Meticulous extraoral and intraoral examinations along with radiographs help in confirming the diagnosis of cherubism, prompting an early diagnosis and management of the disease.
